# Supplementary material for: Anticodon table of the chloroplast genome and identification of putative quadruplet anticodons in chloroplast tRNAs
Source: Sci Rep. 2023 Jan 14;13:760. doi: 10.1038/s41598-023-27886-9 (PMC9840617; doi:10.1038/s41598-023-27886-9)
Supplement: Supplementary file 1 — Supplementary Information 1. [file 41598_2023_27886_MOESM1_ESM.pptx]

## Slide 1
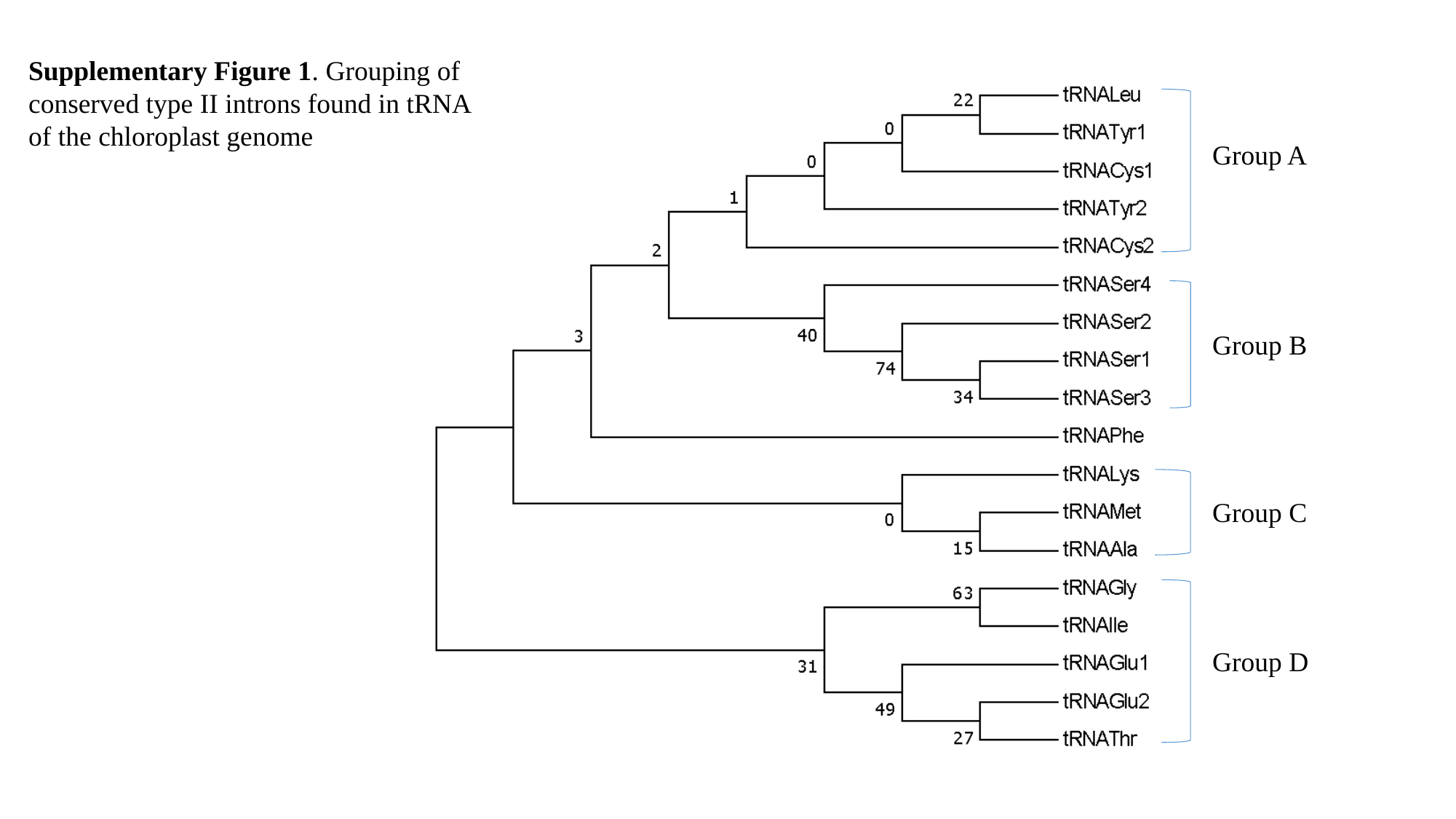

Supplementary Figure 1. Grouping of conserved type II introns found in tRNA of the chloroplast genome
Group A
Group B
Group C
Group D
